# Effect of the masseter muscle injection of botulinum toxin A on the mandibular bone growth of developmental rats

**DOI:** 10.1186/s40902-018-0146-4

**Published:** 2018-03-25

**Authors:** Hyun Seok, Seong-Gon Kim, Min-Keun Kim, Insan Jang, Janghoon Ahn

**Affiliations:** 1 Department of Oral and Maxillofacial Surgery, Chungbuk National University Hospital, Cheongju, 28644 South Korea; 2 Department of Oral and Maxillofacial Surgery, College of Dentistry, Gangneung-Wonju National University, 7 Jukhyun-gil, Gangneung, 25457 South Korea; 3 Department of Orthodontics, College of Dentistry, Gangneung-Wonju National University, Gangneung, 25457 South Korea; 4 Department of Dentistry, College of Medicine, Hallym University, Chuncheon, 24252 South Korea

**Keywords:** Botulinum toxin A, Masseter muscle, Mandible, Growth

## Abstract

**Background:**

The objective of this study was to evaluate the influence of masticatory muscle injection of botulinum toxin type A (BTX-A) on the growth of the mandibular bone in vivo.

**Methods:**

Eleven Sprague-Dawley rats were used, and BTX-A (*n* = 6) or saline (*n* = 5) was injected at 13 days of age. All injections were given to the right masseter muscle, and the BTX-A dose was 0.5 units. All of the rats were euthanized at 60 days of age. The skulls of the rats were separated and fixed with 10% formalin for micro-computed tomography (micro-CT) analysis.

**Results:**

The anthropometric analysis found that the ramus heights and bigonial widths of the BTX-A-injected group were significantly smaller than those of the saline-injected group (*P* < 0.05), and the mandibular plane angle of the BTX-A-injected group was significantly greater than in the saline-injected group (*P* < 0.001). In the BTX-A-injected group, the ramus heights II and III and the mandibular plane angles I and II showed significant differences between the injected and non-injected sides (*P* < 0.05). The BTX-A-injected side of the mandible in the masseter group showed significantly lower mandibular bone growth compared with the non-injected side.

**Conclusion:**

BTX-A injection into the masseter muscle influences mandibular bone growth.

## Background

Botulinum toxin type A (BTX-A) is a bacterial neurotoxin produced by the gram-positive bacterium *Clostridium botulinum* [1]. BTX-A inhibits the release of neurotransmitter in cholinergic nerve terminals and degrades the synaptosomal-associated protein of 25 kDa (SNAP-25) required for acetylcholine fusion and release [2]. It blocks the release of acetylcholine in the presynaptic membranes of neuromuscular junctions and induces reversible muscle weakness and paralysis [3]. BTX-A was first used for the treatment of blepharospasm and strabismus in 1989 and is approved by the US Food and Drug Administration (FDA) [4]. BTX-A has been widely used for the treatment of sialorrhea, facial spasm, and localized muscle hyperactivity, as well as for cosmetic purposes [5, 6]. BTX-A is also administered into the masticatory muscle for the treatment of temporomandibular disorder, bruxism, masticatory myalgia, and masseter muscle hypertrophy [3, 7]. BTX-A can be safely used with few complications, such as bruising, edema, and reversible undesirable muscle paralysis due to diffusion [8].

Mandibular bone morphology can be affected by the masticatory muscle function and activity [9]. In functional matrix theory, craniofacial growth could be regulated by the surrounding soft tissue and muscle function [10]. In human studies, masticatory muscle size and activity are especially correlated with facial bone structure [11, 12]. Masticatory muscle hyperactivity increases the loading of the jaw, leading to increased skeletal bone growth and size [9]. It also increases the bone remodeling rate and bone mineral density, resulting in the increase in mandible size and dental arch width and length [13]. In previous animal studies, masticatory muscle hypofunction by soft diets and muscle or motor nerve resection has induced changes in the mandibular growth and direction [14–16]. However, resection of the muscle and nerve degeneration could induce tissue damage and scar formation [17]. In contrast to these methods, BTX-A injection into the masticatory muscles as a non-invasive procedure can be easily performed and can induce temporary muscle paralysis and weakness.

BTX-A can be selectively administered in the masticatory muscles and can decrease muscle activity without surrounding tissue damage [18]. In a previous study, BTX-A was injected into the masseter and temporalis muscles for evaluating the effect of muscle hypofunction on craniofacial structure changes [19, 20]. Unilateral masseter muscle injection of BTX-A decreased the bone thickness and mineral contents of the ipsilateral side [21], and it could induce mandible asymmetry with the injection side [19]. Further, BTX-A injection into the unilateral masseter and temporalis muscles induced bone loss in the alveolar and condyle regions [20]. BTX-A injection into the unilateral masticatory muscles affects mandibular bone composition, structure, and morphology in adult rats [21]. There are several publications on the effects of BTX-A on mandibular bone growth in growing animals. Many studies have been done in animals greater than 4 weeks of age. In these studies, there should be minimal effect on growth because of the animal’s age. Some studies still claimed that BTX injection in these animals had some effect on growth. However, they injected BTX in the surgically exposed masseter muscle. In these cases, it was unclear whether the growth retardation was due to BTX injection or scar formation in the wound area.

In the present study, we evaluated how the BTX-A injection into the masseter muscles influenced the growth of the mandibular bone in a growing rat model. We measured the anthropometric points and linear distances in the craniofacial bones and compared the changes in bone structure. In this study, we hypothesized that BTX-A injection into the unilateral masseter muscle would induce lower growth rate and a deviated mandible. Thus, the purpose of this study was to evaluate the effects of muscle hypofunction due to BTX-A injection on mandibular bone growth.

## Methods

### Animals’ experiments and study design

Eleven Sprague-Dawley rats were used in this study. The rats were randomly divided into the control and the experimental groups. At 13 days of age, BTX-A or saline was injected into the masseter muscle. The BTX-A (Botulax® 50, botulinum toxin type A, HUGEL, Chuncheon, Korea) was prepared and diluted with 50 mL of saline. BTX-A (0.5 unit) in a 0.5-mL dose was injected intramuscularly into the right masseter muscle in the experimental group (*n* = 6). The same amount of saline was injected into the right masseter muscle in the control group (*n* = 5). The administration of saline and BTX-A was performed at the same time in both groups. All the rats were euthanized at 60 days of age (47 days after injection). The skulls of the rats were separated and fixed with 10% formalin for micro-computed tomography (micro-CT) analysis. This study was approved by the Institutional Animal Care and Use Committee of the Gangneung-Wonju National University, Gangneung, Korea (IACUC GWNU-2016-24).

### Micro-CT analysis

The skulls of rats were assessed by an animal positron emission tomography (PET)/CT/single photon emission computed tomography (SPECT) system (INVEON™, Siemens, Malvern, PA, USA) at the Ochang Center at the Korea Basic Science Institute. The CT scanner was set to an 80 KV voltage for the X-ray tube, 500 μA current for the X-ray source, and 210 ms of exposure time. The detector and the X-ray source were rotated 360 degrees in 360 rotation steps. The number of calibration exposures was 30. The system magnification was allowed over 30.7 mm of the axial field of view (FOV) and 30.7 mm of the transaxial FOV. The scanned images were reconstructed with an Inveon Research Workplace Software (Siemens Healthcare). The calibrated three-dimensional (3D) images were shown in gross profiles of the skulls of the rats.

### Anthropometric measurement points and distances

**Fig. 1:**
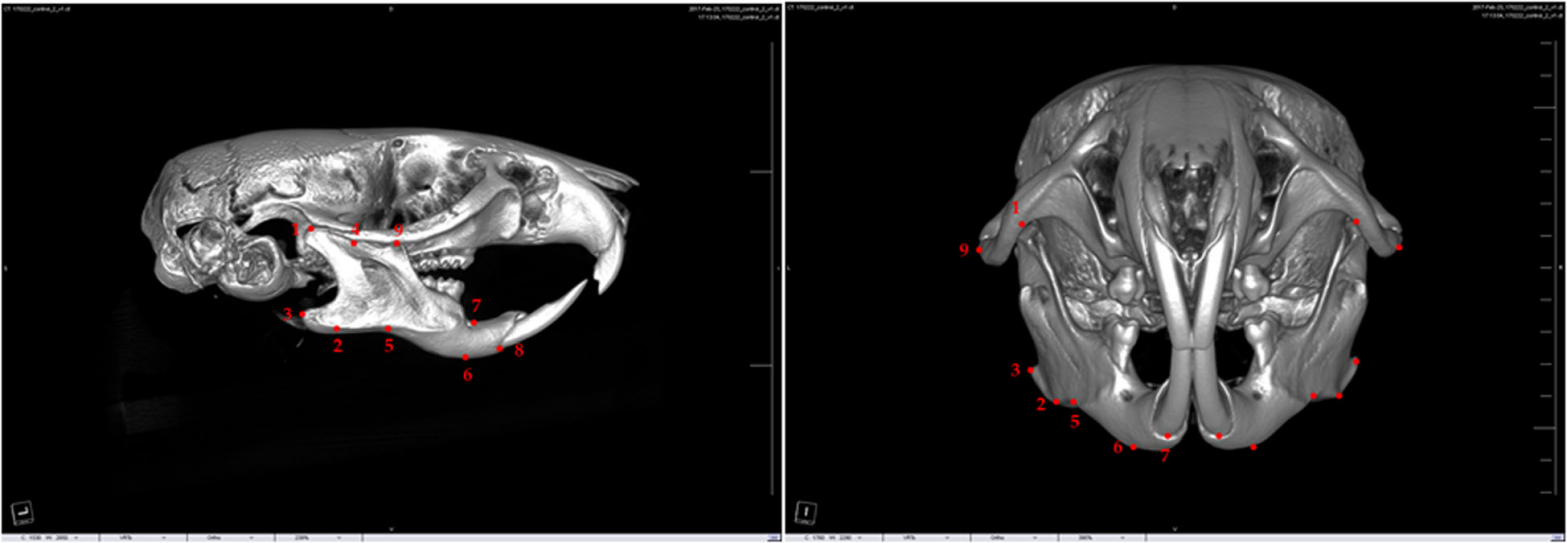
Anthropometric measurement points. (1) Condylion (Cd)—most posterior and superior points on the mandibular condyle. (2) Gnathion (Gn)—most inferior point of the bony contour of the gonial angle of the mandible. (3) Gonion (Go)—most posterior point of the bony contour of the gonial angle of the mandible. (4) Coronoid notch (Co)—most inferior point of the coronoid notch. (5) Antegonial notch—most superior point of the curvature of the antegonial notch. (6) Menton (Me)—most inferior point of the mandibular symphysis. (7) Mandibular alveolar point—the deepest point on the mandibular alveolar crest between the lower incisor and the lower first molar. (8) Infradentale—most inferior point of the marginal alveolar bone of the lower central incisor. (9) Zygion (Zy)—most external point of the zygomatic arch

**Fig. 2:**
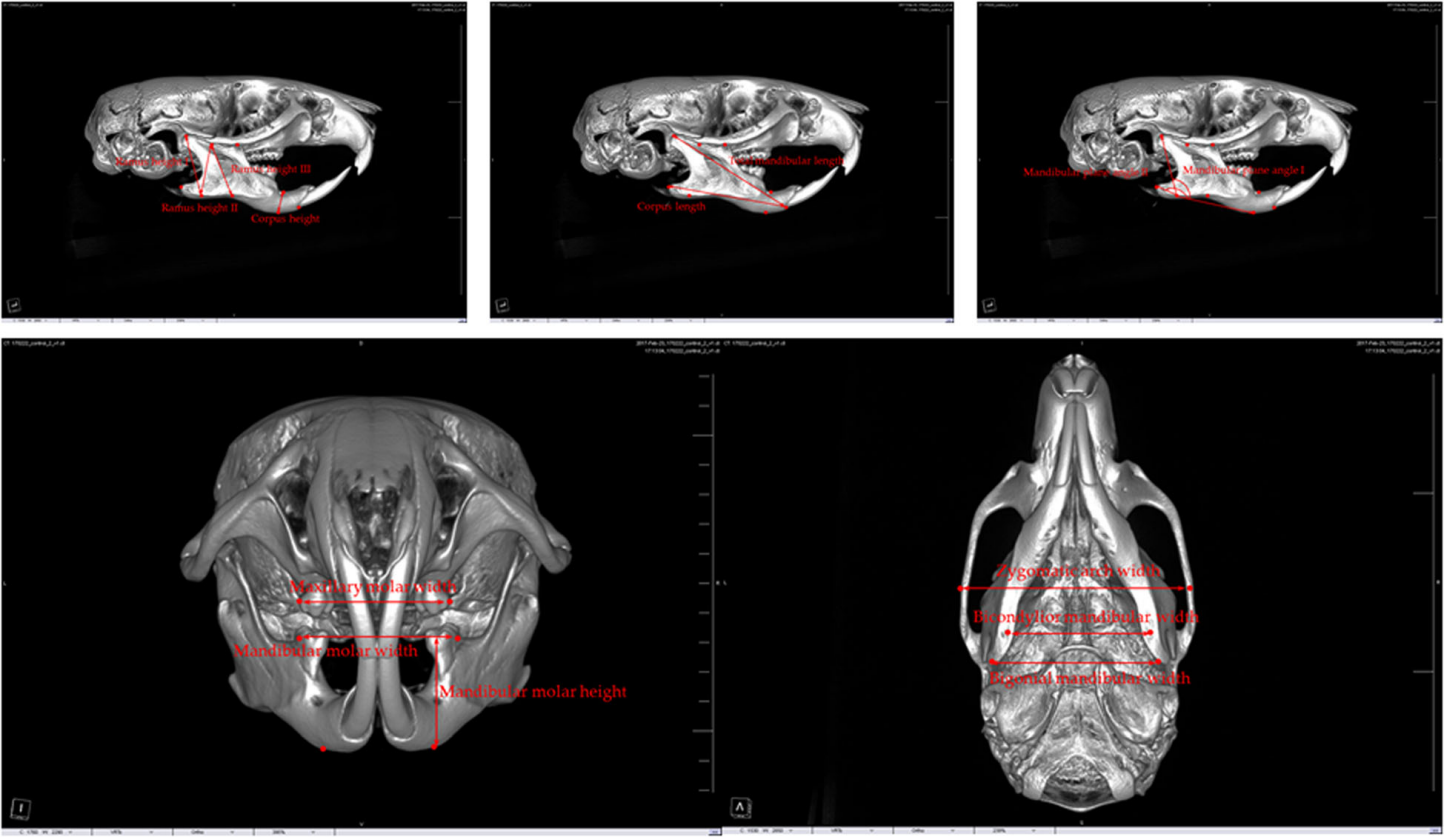
Anthropometric measurement of the rat maxillofacial bone on micro-computed tomography images. Each measurement is explained in Table 1

Anthropometric points were identified, and the distance was measured for the evaluation of mandibular growth changes on micro-CT images. Anthropometric points are shown and described in Fig. 1. A total of nine anthropometric points were chosen: condylion, gnathion, gonion, coronoid notch, antegonial notch, menton, mandibular alveolar point, infradentale, and zygion. The linear distance of each point was measured on vertical, sagittal, and transverse planes, and the definition of each distance is explained in Table 1. Each measurement was shown in Fig. 2.

**Table 1:** The definition of each anthropometric measurement. The linear distances of each anthropometric point measured on the vertical, sagittal, and transverse planes

Variables	Definition
Vertical measurement	
Ramus height I	Distance between the condylion and gnathion
Ramus height II	Distance between the coronoid notch and gnathion
Ramus height III	Distance between the coronoid notch and antegonial notch
Corpus height	Distance between the mandibular alveolar point and menton
Mandibular molar height	Distance between the mesiobuccal cusp of the mandibular first molar and menton
Sagittal measurement	
Mandibular plane angle I	Angle between the line connecting the condylion and gnathion and the line connecting the gnathion and menton
Mandibular plane angle II	Angle between the line connecting the gonion and gnathion and the line connecting the gnathion and menton
Total mandibular length	Distance between the condylion and infradentale
Corpus length	Distance between the gonion and infradentale
Transverse measurement	
Zygomatic arch width	Distance between the bilateral zygions
Maxillary molar width	Distance between the bilateral mesiobuccal cusps of the maxillary first molars
Mandibular molar width	Distance between the bilateral mesiobuccal cusps of the mandibular first molars
Bicondylior mandibular width	Distance between the bilateral condylions
Bigonial mandibular width	Distance between the bilateral gonions
Dental midline discrepancy	Distance between the lower incisor midline and the upper incisor midline

### Statistical analysis

When there were two compared groups and the comparisons were performed in the samples within the same animal, a paired *t* test was used. For the comparison of the control group and the experimental group, an independent sample *t* test was used. Differences with *P* values less than 0.05 were considered significant.

## Results

### Comparison of the BTX-A-injected group and the saline-injected group

**Fig. 3:**
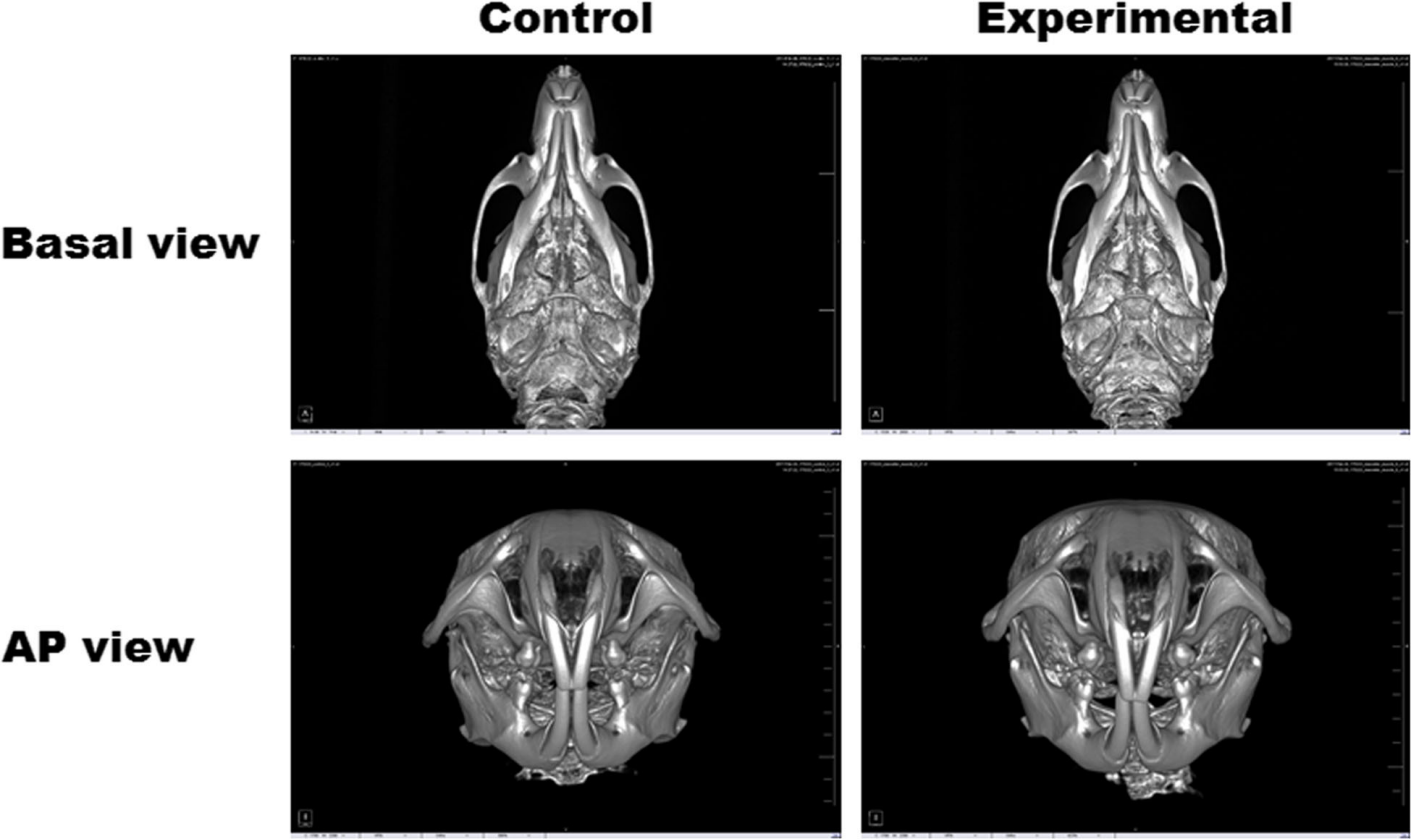
Micro-computed tomography of rats. Basal view and anteroposterior view are shown for the control group (saline-injected) and the experimental group (BTX-A-injected)

Most of the average vertical anthropometric measurements of the mandibular bones in the experimental (BTX-A-injected) group were less than in the control (saline-injected) group (Table 2, Fig. 3). The average measurements of ramus height I were 5.39 ± 0.21 and 5.10 ± 0.12 mm in the control group and in the experimental group, respectively. The average measurements of ramus height III were 4.51 ± 1.58 and 3.72 ± 0.17 mm in the control group and the experimental group, respectively. There were significant differences in ramus heights I and III between groups (*P* = 0.025 and 0.002, respectively). The average total mandibular and corpus lengths in the experimental group were less than in the control group (Table 2). The average measurements of mandibular plane angle II were 157.13 ± 5.84 and 163.78 ± 6.07° in the control group and the experimental group, respectively (*P* = 0.008).

**Table 2:** Comparison of anthropometric measurements of the maxillofacial bone

Variables	Control	Experimental	*P* value
	Mean ± SD	Mean ± SD	
Vertical measurement (mm)			
Ramus height I	5.39 ± 0.21	5.10 ± 0.12	0.025*
Ramus height II	4.10 ± 0.16	3.91 ± 0.15	NS
Ramus height III	4.51 ± 1.58	3.72 ± 0.17	0.002*
Corpus height	2.67 ± 0.72	2.43 ± 0.28	NS
Mandibular molar height	3.97 ± 0.06	3.92 ± 0.12	NS
Sagittal measurement			
Mandibular plane angle I (°)	112.18 ± 2.65	113.47 ± 2.86	NS
Mandibular plane angle II (°)	157.13 ± 5.84	163.78 ± 6.07	0.008*
Total mandibular length (mm)	11.74 ± 0.19	11.58 ± 0.35	NS
Corpus length (mm)	10.43 ± 0.24	10.39 ± 0.21	NS
Transverse measurement (mm)			
Zygomatic arch width	12.31 ± 0.30	11.97 ± 0.33	NS
Maxillary molar width	3.98 ± 0.26	4.04 ± 0.18	NS
Mandibular molar width	4.39 ± 0.12	4.35 ± 0.25	NS
Bicondylior mandibular width	10.47 ± 0.21	10.27 ± 0.22	NS
Bigonial mandibular width	9.94 ± 0.21	9.26 ± 0.15	< 0.001*
Dental midline discrepancy (lower incisor midline right: +, left: −)	− 0.032 ± 0.090	0.035 ± 0.118	NS

*NS* not significant

*P < 0.05

For the transverse measurements, only the bigonial mandibular width was significantly different between the saline-injected group and the BTX-A-injected group (*P* < 0.001). The dental midline of the lower anterior tooth compared with the upper tooth was deviated to the right side by 0.035 ± 0.118 mm in the experimental group.

### Comparison of the vertical and sagittal anthropometric measurements between the right and left sides of the mandibular bone in the BTX-injected group

There was no significant difference between the right and left sides of mandibular bone growth in the saline-injected group (Fig. 3). In the BTX-A-injected group, the average vertical measurements of ramus height I, II, and III; total mandibular length; and corpus length were less in the right (BTX-A injection) side than in the left side (Table 3, Fig. 3), and there were significant differences in the ramus height II and III (*P* = 0.012 and 0.040, respectively). The average sagittal measurements of mandibular plane angles I and II were greater on the right side (BTX-A injection) than on the left side. There were significant differences in the mandibular plane angles I and II (*P* = 0.006 and 0.007, respectively).

**Table 3:** Comparison of anthropometric measurements of the maxillofacial bone between the right and left sides in the experimental group (BTX-A injection in the right masseter muscle)

Variables	Right side (BTX-A)	Left side	*P* value
	Mean ± SD	Mean ± SD	
Vertical measurement (mm)			
Ramus height I	5.09 ± 0.11	5.11 ± 0.15	NS
Ramus height II	3.79 ± 0.18	4.04 ± 0.22	0.012*
Ramus height III	3.68 ± 0.20	3.75 ± 0.15	0.040*
Corpus height	2.43 ± 0.28	2.43 ± 0.28	NS
Mandibular molar height	3.95 ± 0.12	3.89 ± 0.12	NS
Sagittal measurement			
Mandibular plane angle I (°)	114.94 ± 3.04	111.99 ± 4.09	0.006*
Mandibular plane angle II (°)	167.42 ± 8.48	160.14 ± 7.29	0.007*
Total mandibular length (mm)	11.51 ± 0.40	11.65 ± 0.32	NS
Corpus length (mm)	10.37 ± 0.18	10.42 ± 0.25	NS

*NS* not significant

*P < 0.05

## Discussion

Mandibular bone growth can be affected by masticatory muscle function and activity [20]. Hyperactivity of the masticatory muscle function can contribute to the increases in mandibular bone growth, size, and structure [22]. Conversely, reduced muscle function can lead to the low growth of the craniofacial bone in the rat model [23]. In the present study, we evaluated the effects of masseter muscle hypofunction due to BTX-A injection on the growth of the mandibular bone in vivo. In anthropometric measurements, the BTX-A-injected rats showed a low growth of the mandibular bone compared with that of the saline-injected rats (Table 2, Fig. 3). The experimental group showed decreased ramus heights I and III and bigonial mandibular width and increased mandibular plane angle II (Table 2). The dental midline of the lower anterior tooth was slightly deviated to the injection side (Fig. 3). Comparing the BTX-A injection side and non-injected side, the experimental group showed decreased ramus heights II and III and increased mandibular plane angles I and II on the BTX-A-injected side (Table 3). Consequently, the masseter muscle hypofunction due to the BTX-A injection contributed to the mandibular bone growth and bony morphology.

BTX-A could decrease muscle activity and show paralytic effects immediately after injection in a rabbit model [18]. The maximum paralytic effect of BTX-A appeared at 2 to 3 weeks after injection [18], and this effect persisted for approximately at 4 to 6 weeks in rodents [24]. For the evaluation of the effect of muscle hypofunction on skeletal bone growth, BTX-A should be administered to the target muscle before the beginning of muscle and bone development. Generally, rats experience puberty at 35 days after birth, and skeletal bone maturity is attained at 60 days of age [25]. In our experiment, we performed BTX-A injection in 13-day-old rats before the skeletal development completion. In several animal studies, the administration of BTX-A has been performed at 30 days after birth [17, 19, 22, 25]. In this study, we administered BTX-A as soon as possible considering the period of time needed to attain the maximum paralytic effect, and the anthropometric measurements were analyzed at 60 days of age, after skeletal bone maturity. During this period, paralysis of the masticatory muscles was achieved, and it affected the mandibular bone growth of rats.

BTX-A can be safely used in the mandibular region because only a small amount of BTX-A is required for the treatment of the muscle disorder or for cosmetic procedures, compared with spasticity treatment [26, 27]. The dosage of BTX-A in the masticatory muscles varies according to the age and body weight in vivo [22, 23]. In this experiment, the administration dose of BTX-A was determined by our preliminary study. The 0.5 units of BTX-A were administered to the masseter and temporalis muscles in 13-day-old rats [28], whereas the lethal dose of BTX-A in rats ranges from 50 to 200 units/kg [29]. In our preliminary study, very young rats did not sustain the high doses of BTX-A administration within the lethal dose (data not shown). Therefore, 0.5 units of BTX-A was used to paralyze the target muscles considering our experience and previous report [28].

Decreased muscle activity can affect the bone metabolism and remodeling processes [30]. The diminished muscle force induces bone osteoclastic processes and disrupts bone homeostasis [31]. Paralysis of the muscle contributes to bone degradation and changes in the bone morphology [30]. When BTX-A is injected into the masseter muscle, the mandibular bone mineral content is significantly reduced [21], and the thickness of the mandible ramus is also decreased with masseter muscle atrophy [32]. Paralysis of the masseter and temporalis muscles induces bone loss in the alveolar and condylar areas [20]. In this study, the ramus height and bigonial mandibular width in the experimental group were significantly less than in the control group (Table 2), and the experimental group had a larger mandibular plane angle than the control group. This result could be explained by the low growth of the mandible angle area, to which the masseter muscle is attached. Further, hypoplasia of the mandible angle area led to reduced ramus height and bigonial mandibular width and an increased mandibular plane angle. This morphological bone change is in accord with the functional matrix theory that the soft tissue and muscle activity are major contributing factors in the mandibular bone growth. From these results, we could confirm that the hypofunction and paralysis of the masseter muscle induced low growth and changes in the morphology of the mandible.

BTX-A injection into the masseter muscles before bone development caused the low growth of the mandibular bone [19, 22, 23]. Most anthropometric measurements showed less growth on the BTX-A injection side, compared with saline injection or the non-injected side (Tables 2 and 3). In the case of saline injection, there was no significant difference between the injected side and the non-injected side (data not shown). Ramus heights II and III were significantly smaller in the BTX-injected side compared with those in the non-injected side (*P* < 0.05; Table 3). However, the effect of BTX injection on the growth was small compared with the effects of myomectomy and motor nerve denervation [16, 17]. The differences in the dental midline discrepancy were within 1 mm between the groups (Table 2). In a previous study, BTX-A injection into the unilateral masseter muscle in 4-week-old rats induced severe facial asymmetry with dental midline deviation to the ipsilateral side [19]. However, in the previous study, the administration of BTX-A was performed after surgical exposure of the masseter muscle [19]. Surgical exposure provides greater visibility of the injection site. However, this surgical procedure could produce scar tissue formation and muscle atrophy, and it can lead to a severely asymmetric mandible. In our experiment, we administered BTX-A by intramuscular injection to prevent other etiological factors from affecting the bone morphology. Unilateral injection of BTX-A induces changes in the bony morphology and structure regardless of age [21, 22, 28]. Our results showed the effects of BTX-A injection on the low growth of the mandible. However, BTX-A injection alone does not cause significant bony changes and severe mandible asymmetry.

The influence of paralytic effects of BTX-A on the mandibular bone growth is a clinically valuable result. BTX-A has been used for facial muscle disorders, TMJ problems, and cosmetic procedures [7]. Recently, BTX-A has been diversely used in the mandibular regions for the treatment of post-traumatic complications and orthognathic surgery [33, 34]. BTX-A injection into the anterior belly of the digastric muscle has been used for the correction of anterior open bite in bi-angle mandible fracture patients [33, 35], and BTX-A into the lower lip depressor muscle was used for the treatment of an asymmetric lower lip [36]. The masseter muscle function can affect the instability of the displaced mandible segments after orthognathic surgery [36]. Hypofunction of the masticatory muscle can contribute to stability post-orthognathic surgery [33]. The effect of BTX-A on the mandibular bone growth has been the subject of debate. Previous animal studies have shown less growth of the mandibular bone by BTX-A administration in vivo [21, 28]. However, the degree of bone morphological changes due to BTX-A has varied across studies [19]. BTX-A injection into the masticatory muscle during growth could induce low growth of the mandible and facial asymmetry. However, the degree of asymmetric change in our study was insufficient to induce severe facial asymmetry, dental midline deviation, or dental malocclusion.

## Conclusion

In this study, we evaluated the paralytic effects of BTX-A on the mandibular bone growth in vivo. The injection of BTX-A into the masseter muscle was performed in 13-day-old rats. Anthropometric measurement analysis showed that the BTX-A-injected group had significantly decreased ramus heights I and III and bigonial mandibular width (*P* = 0.025, 0.002, and 0.006) and increased mandibular plane angle II, compared with the saline-injected group (*P* < 0.001). Further, the BTX-A-injected side had significantly smaller ramus height and significantly higher mandibular plane angle, compared with those of the non-injected side (*P* < 0.05). From these results, we could confirm that BTX-A injection into the masseter muscle during the growth phase could cause low growth of the mandibular bone. This animal study was useful for evaluating the degree of the low skeletal bone growth effects of BTX-A. Anthropometric evaluations showed that BTX-A injection affected the bony growth pattern.

## Abbreviation

BTX-A: Botulinum toxin A
